# The influence of parental educational involvement on learning engagement among first-year college students: the mediating effects of academic self-efficacy and professional identity

**DOI:** 10.3389/fpsyg.2025.1738085

**Published:** 2026-01-12

**Authors:** Yufen Zhao, Yao Qin, Liang Zhang

**Affiliations:** 1 College of Education for the Future, Beijing Normal University, Zhuhai, China; 2 Educational Teaching Research Center, Handan University, Handan, China; 3 School of Fine Arts and Design, Handan University, Handan, China; 4 College Students’ Mental Health Education Center, Northeast Agricultural University, Harbin, China

**Keywords:** first-year students, learning engagement, parental educational involvement, professional identify, self-efficacy

## Abstract

This study examined how parental educational involvement influences learning engagement among first-year college students and tested the mediating roles of academic self-efficacy and professional identity. Using cluster sampling, survey data were collected from 803 first-year undergraduates (228 male, 575 female, with average of 18 years). Serial mediation was tested using PROCESS Model 6 with 5,000 bootstrap resamples. Parental educational involvement positively predicted learning engagement, and the direct effect remained significant after including the mediators (*β* = 0.14, *p* < 0.001), indicating partial mediation. The overall regression model explained substantial variance in learning engagement (*R*^2^ = 0.37; *F* = 155.46, *p* < 0.001), and the total indirect effect accounted for 57.54% of the total effect. These findings suggest that parental educational involvement continues to shape students’ engagement in the first year of university, primarily through strengthening students’ competence beliefs and professional identification.

## 1 Introduction

In 2000, the United States comprehensively launched an evaluation project that used college students’ learning engagement as an indicator of higher educational teaching quality, thereby initiating the National Survey of Student Engagement (NSSE) to promote reforms in higher educational teaching quality (Kandiko Howson and Matos, 2021). The implementation of this project sparked widespread attention within the higher educational community toward college students’ learning engagement, and the use of engagement as a measure of teaching quality has since been extensively adopted worldwide. Numerous empirical studies have confirmed that learning engagement is a key factor influencing college students’ academic achievement (Luo et al., 2023; Lin et al., 2023; Qureshi et al., 2023). Over the past two decades, in order to enhance college students’ learning engagement, researchers have successively identified various factors such as the school environment, teacher support, and teaching strategies that influence students’ learning engagement (Lowe and El Hakim, 2020; Chi, 2017). However, relatively few studies have focused on the influence of family educational factors on college students’ learning engagement, as researchers generally believe that parental educational involvement plays a more significant role during basic educational.

However, some studies have confirmed that parental educational involvement continues to play an important role in college students’ academic life (Smojver-Azic et al., 2016). Particularly within the Chinese cultural context, President Xi Jinping has repeatedly emphasized the importance of cultivating good family values, and the promulgation of the *Family Educational Promotion Law* further reflects the lasting impact and significance of family educational on an individual’s lifelong development. Therefore, investigating the parental educational involvement on college students’ learning engagement holds substantial theoretical and practical importance.

In the course of college students’ academic life, the first and fourth years are the key stages that influence the quality of talent cultivation, particularly the first-year stage (Nabil et al., 2021). Upon entering a new learning environment, changes in social support systems make it difficult for first-year students to quickly and comprehensively engage in high-quality learning (Wang et al., 2024). Emotional support and cognitive encouragement from parents can effectively alleviate first-year students’ learning anxiety (Liu H. et al., 2025), enhance their academic self-efficacy, strengthen their professional identity, and thereby promote their learning engagement (Yuan et al., 2016). The learning engagement of first-year students plays an important role throughout their academic career and directly affects the quality of learning in the following 4 years. Therefore, this study takes first-year college students as its research subjects, aiming to examine the influence mechanism of parental educational involvement on students’ learning engagement at the initial stage of higher educational and to explore the indirect effects within this process.

### 1.1 Parental educational involvement and learning engagement among first-year college students

Parental educational involvement refers to the various educational behaviors parents undertake at home and in school to promote their children’s academic and psychological development (Shao et al., 2022). Parental educational involvement can enhance students’ school performance and promote their learning engagement.

Learning engagement refers to the degree of effort and energy individuals invest in the learning process. Some studies conceptualize learning engagement as consisting of behavioral, cognitive, and emotional dimensions (Liu H. et al., 2025; Lowe and El Hakim, 2020), and measure the quality of engagement based on these dimensions. Other scholars regard learning engagement as a positive and persistent mental state exhibited during learning, emphasizing the psychological vitality of the process. Accordingly, they assess learning engagement through three dimensions: vigor, dedication, and absorption (Abdul Jabbar et al., 2025). According to Self-Determination Theory, individuals can enhance their intrinsic motivation by satisfying three basic psychological needs, autonomy, competence, and relatedness through the social environment they inhabit (Liu et al., 2014; Chi, 2017). In other words, positive attention and emotional support from significant others can effectively stimulate students’ enthusiasm and vitality for learning. Parental educational involvement often includes emotional support that strengthens students’ emotional skills and well-being, the psychological resources that sustain motivation and translate into higher learning engagement (Sorrenti et al., 2024).

Therefore, this study proposes:

Hypothesis 1: Parental educational involvement positively predicts first-year college students’ learning engagement.

### 1.2 The mediating role of academic self-efficacy

Academic self-efficacy refers to an individual’s subjective judgment of their own ability to successfully complete specific learning tasks (Bandura, 1993). A higher level of academic self-efficacy can effectively promote students’ learning engagement (Meng and Zhang, 2023). Bandura proposed that an individual’s cognitive development is jointly influenced by social interaction and self-efficacy. His Social Cognitive Theory confirmed the mechanism through which social interaction influences cognitive development (Bandura, 1977). According to this perspective, cognitive development is shaped by interactions within the social environment, such as role models and social support (Sorrenti et al., 2025). For instance, students’ perceptions of parental modeling and support are positively correlated with their academic self-efficacy (Upadyaya and Salmela-Aro, 2013; Turner et al., 2009). Moreover, parents’ positive emotional encouragement and support can effectively enhance students’ self-efficacy, thereby improving their learning engagement (Yuan et al., 2016).

Therefore, this study proposes:

Hypothesis 2: Academic self-efficacy mediates the relationship between parental educational involvement and first-year college students’ learning engagement.

### 1.3 The mediating role of professional identity

Professional identity refers to the emotional acceptance and recognition that individuals develop based on their understanding and awareness of their academic major, which motivates them to explore it further with a positive attitude and proactive behavior (Sarraf-Yazdi et al., 2021). A strong sense of professional identity among college students can positively influence their learning engagement (Liao et al., 2024). This effect is particularly significant during the first year of college when students transition from unfamiliarity to deeper understanding; if they develop a positive professional identity, they are more likely to engage actively in their academic studies.

According to Self-Determination Theory, when individuals make autonomous choices based on a thorough understanding of their personal needs and environmental information, such actions foster positive self-improvement (Goldman et al., 2017). In this sense, professional identity promotes students’ autonomous learning behaviors. Parental educational guidance can help first-year students gain an early and comprehensive understanding of their academic major, thereby enhancing their professional identity and subsequently influencing their learning engagement.

Therefore, this study proposes:

Hypothesis 3: Professional identity mediates the relationship between parental educational involvement and first-year college students’ learning engagement.

### 1.4 The mediating role of academic self-efficacy and professional identity

Both academic self-efficacy and professional identity are important factors influencing college students’ learning engagement. Previous studies have demonstrated that self-efficacy in learning and professional identity each positively predict students’ learning engagement. However, it remains unclear whether parental educational involvement affects learning engagement through the combined sequential influence of these two factors, as existing research has not yet verified this relationship.

Nevertheless, based on Self-Determination Theory and Social Cognitive Theory, it can be inferred that parental educational involvement, as an important environmental factor in college students’ external context, can influence their academic self-efficacy and professional identity, which in turn affect their learning engagement.

Therefore, this study proposes:

Hypothesis 4: Academic self-efficacy and professional identity play a chain mediating role between parental educational involvement and first-year college students’ learning engagement.

Based on existing theories and relevant empirical findings, parental educational involvement has been shown to exert a significant influence on children’s and early adolescents’ learning engagement; however, its effect during late adolescence and early adulthood remains unclear. Furthermore, both academic self-efficacy and professional identity have been found to positively predict college students’ learning engagement, yet whether these two variables serve as significant mediators in the relationship between parental educational involvement and first-year college students’ learning engagement requires further investigation.

Therefore, grounded in Self-Determination Theory and Social Cognitive Theory, this study aims to explore the relationship between parental educational involvement and first-year college students’ learning engagement, as well as to examine the mediating roles of academic self-efficacy and professional identity within this relationship.

## 2 Methodology

### 2.1 Participants

This study adopted a cluster sampling method to conduct a questionnaire survey among first-year students from 12 majors at a local undergraduate university. A total of 840 questionnaires were distributed in classrooms by major, and 820 valid responses were collected, yielding a response rate of 97.62%. After eliminating questionnaires with duplicate or incomplete responses, 803 valid questionnaires were retained for data analysis. Among the participants, 228 were male and 575 were female, with average of 18 years; 356 were enrolled in humanities and social sciences, 333 in science and engineering, and 114 in arts and sports disciplines.

### 2.2 Research instruments

#### 2.2.1 Parental educational involvement scale

The Parental Educational Involvement Scale developed by Song (2010) was adopted for this study. The original scale consists of three dimensions: emotional involvement, intellectual involvement, and behavioral involvement. Considering that after entering university, the behavioral involvement of parents has relatively limited influence on first-year students, this study utilized only the emotional and intellectual involvement dimensions.

The wording of several items was appropriately modified to fit the characteristics of college students, resulting in a total of 26 items retained in the final version. The scale employs a 5-point Likert scoring system, where higher scores indicate a higher level of parental educational involvement. In this study, the Cronbach’s *α* coefficients for the two subscales and the total scale were 0.92, 0.92, and 0.95, respectively.

#### 2.2.2 Learning engagement scale (UWES-S)

The Learning Engagement Scale (UWES-S) developed by Schaufeli et al. (2002) and translated and revised was used in this study. The scale comprises three dimensions: vigor, dedication, and absorption, with a total of 17 items. It employs a 5-point Likert scale, where higher scores indicate a higher level of learning engagement. The Cronbach’s *α* coefficients for the three subscales and the total scale were 0.88, 0.87, 0.88, and 0.95, respectively.

#### 2.2.3 Professional identity scale

The Professional Identity Scale developed by Qin (2009) was adopted to assess students’ professional identity. This scale consists of four dimensions: cognitive, emotional, behavioral, and adaptive, comprising a total of 23 items. It uses a 5-point Likert scale, with higher scores indicating a higher level of professional identity. The Cronbach’s *α* coefficients for the four subscales and the total scale were 0.90, 0.93, 0.88, 0.87, and 0.96, respectively.

#### 2.2.4 Academic self-efficacy scale

The Academic Self-Efficacy Scale originally developed by Pintrich and De Groot (1990) and translated and revised by Liang (2000) was used to measure students’ perceptions of their learning competence and behavior. The scale includes two dimensions: self-efficacy for learning ability and self-efficacy for learning behavior, with a total of 22 items. It adopts a 5-point Likert scale, where higher scores represent a higher level of academic self-efficacy. The Cronbach’s *α* coefficients for the two subscales and the total scale were 0.95, 0.95, and 0.97, respectively.

After obtaining informed consent from all participants, the questionnaire was administered on a voluntary basis. Data collection was conducted collectively in classrooms, organized by major. Trained administrators read standardized instructions to ensure consistency in administration, distributed the questionnaires uniformly, and collected them on-site immediately after completion.

Data were analyzed using SPSS 25.0. First, descriptive statistics and correlation analyses were performed on all variables. Subsequently, PROCESS macro Model 6 was employed to examine the chain mediating effects among variables. The significance of the mediation effects was tested using the nonparametric percentile Bootstrap method (Hayes, 2018).

## 3 Results

### 3.1 Test for common method bias

To examine potential common method bias, Harman’s single-factor test was conducted. All measurement items were entered into an exploratory factor analysis, which extracted 13 common factors with eigenvalues greater than 1. The first factor explained 20.86% of the total variance, which is below the critical threshold of 40%. This result indicates that there was no serious common method bias in the data.

### 3.2 Descriptive statistics and correlation analysis

Descriptive statistics and correlation analyses were conducted using SPSS 25.0. The results revealed that parental educational involvement, academic self-efficacy, professional identity, and learning engagement were all positively and significantly correlated with one another (*r* = 0.17–0.58, *p* < 0.01). Detailed results are presented in Table 1.

**Table 1:** Descriptive statistics and correlation analysis results.

Variables	*M*	*SD*	1	2	3
1 Parental educational involvement	3.19	0.78	1		
2 Academic self-efficacy	3.99	0.59	0.17^**^	1	
3 Professional identify	3.71	0.58	0.34^**^	0.33^**^	1
4 Learning engagement	3.41	0.65	0.33^**^	0.30^**^	0.58^**^

*N* = 803; ^*^p < 0.05, ^**^p < 0.01, ^***^p < 0.001.

### 3.3 Test of mediation effects

The nonparametric percentile Bootstrap method was employed to test the mediating effects, with 5,000 samples drawn. The results of the regression analyses among the variables are presented in Table 2.

**Table 2:** Regression relationships among variables.

Dependent variable	Independent variable	*R*	*R* ^2^	*F*	*β*	*t*
Learning engagement		0.33	0.11	94.64^***^		
	Parental educational involvement				0.33	9.73^***^
Academic self-efficacy		0.17	0.03	24.27^***^		
	Parental educational involvement				0.17	4.92^***^
Professional identify		0.43	0.19	94.88^***^		
	Parental educational involvement				0.29	8.93^***^
	Academic self-efficacy				0.28	8.79^***^
Learning engagement		0.61	0.37	155.46^***^		
	Parental educational involvement				0.14	4.61^***^
	Academic self-efficacy				0.11	3.68^***^
	Professional identity				0.50	15.96^***^

*p* < 0.001. *N* = 803. All variables were standardized.

Parental educational involvement had a significant positive predictive effect on first-year students’ learning engagement (*β* = 0.33, *p* < 0.001). It also significantly and positively predicted academic self-efficacy (*β* = 0.17, *p* < 0.001) and professional identity (*β* = 0.29, *p* < 0.001). Academic self-efficacy significantly and positively predicted professional identity (*β* = 0.28, *p* < 0.001) and learning engagement (*β* = 0.11, *p* < 0.001). Additionally, professional identity significantly and positively predicted learning engagement (*β* = 0.50, *p* < 0.001).

When both academic self-efficacy and professional identity were included in the model, parental educational involvement still significantly and positively predicted learning engagement (*β* = 0.14, *p* < 0.001), indicating the presence of partial mediation effects.

**Figure 1:**
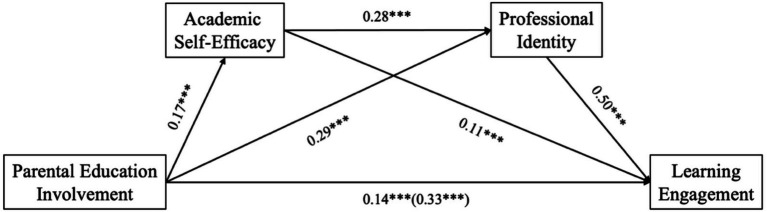
Path diagram for mediating effect test.

As shown in Table 3 and Figure 1, both academic self-efficacy and professional identity exhibited significant mediating effects in the relationship between parental educational involvement and learning engagement. The total standardized indirect effect was 0.187, while the total effect of the model was 0.325, indicating that the overall mediating effect accounted for 57.54% of the total effect.

**Table 3:** Mediation effect analysis results.

Effect	Path	Standardized indirect effect estimates	Effect size	95% confidence interval	
				Lower bound	Upper bound
Direct effect	Parental educational involvement → learning engagement	0.138	42.46%	0.07	0.16
Indirect effects	Parental educational involvement → academic self-efficacy → learning engagement	0.019	5.85%	0.01	0.04
	Parental educational involvement → professional identity → learning engagement	0.144	44.31%	0.11	0.18
	Parental educational involvement → academic self-efficacy → professional identity → learning engagement	0.024	7.38%	0.01	0.04
Total indirect		0.187	57.54%	0.14	0.24
Total effect		0.325		0.22	0.33

Specifically, the indirect effect through the path “Parental Educational Involvement → Academic Self-Efficacy → Learning Engagement” was 0.019, representing 5.85% of the total effect. The indirect effect through the path “Parental Educational Involvement → Professional Identity → Learning Engagement” was 0.144, accounting for 44.31% of the total effect. The indirect effect through the sequential path “Parental Educational Involvement → Academic Self-Efficacy → Professional Identity → Learning Engagement” was 0.024, contributing 7.38% to the total effect.

The 95% confidence intervals for all three indirect paths did not include zero, indicating that the mediating effects were all statistically significant.

## 4 Discussion

Grounded in Self-Determination Theory and Social Cognitive Theory, this study examined the relationship between parental educational involvement and first-year college students’ learning engagement, as well as the chain mediating roles of academic self-efficacy and professional identity in this relationship. The findings provided empirical support for all proposed hypotheses, confirming that parental educational involvement not only exerts a direct positive influence on students’ learning engagement but also indirectly enhances it through the mediating effects of academic self-efficacy and professional identity.

### 4.1 Interpreting the predominance of indirect effects over the direct pathway

Although parental educational involvement still predicts learning engagement directly, the mediation results indicate that its influence operates primarily through students’ internal psychological processes, with the combined indirect pathways exceeding the direct effect. This pattern is theoretically plausible in the first-year university context because parental involvement becomes less able to shape students’ daily learning behaviors through monitoring or direct control; instead, it functions mainly as a developmental resource that strengthens students’ beliefs and self-concepts. Specifically, parental educational involvement may enhance academic self-efficacy by communicating support, expectations, and access to learning resources, which increases students’ perceived capability to handle academic demands (Bandura, 1997). Higher self-efficacy, in turn, facilitates exploration and commitment to one’s academic pathway, strengthening professional identity, which provides meaning, direction, and persistence in learning activities. This sequential process helps explain why the chain pathway is significant: it reflects a progressive mechanism in which confidence (self-efficacy) translates into a more stable role-related self-concept (professional identity), which then energizes sustained engagement. Practically, the findings imply that efforts to increase first-year students’ engagement should prioritize interventions that convert parental support into competence beliefs and identity formation, rather than relying on direct parental involvement alone. Universities can operationalize this by embedding efficacy-building learning supports (e.g., early academic coaching, scaffolded success experiences, timely feedback) alongside identity-focused programming (e.g., major orientation, mentoring with seniors, career narrative activities, discipline-based communities). For parents, the implication is to adopt autonomy-supportive involvement, encouraging goal-setting, offering informational support, and affirming students’ developing professional direction, so that involvement strengthens self-efficacy and professional identity, thereby producing more durable gains in engagement (Oyserman et al., 2021).

### 4.2 The relationship between parental educational involvement and learning engagement

This study found that parental educational involvement was not only significantly correlated with first-year college students’ learning engagement, but also could directly and positively predict their learning engagement. This result verifies Hypothesis 1. Most first-year college students are generally around 17 or 18 years old, which indicates that parental educational involvement still exerts a certain influence on learning engagement during early adulthood. Although the magnitude of this influence is lower than that observed during childhood and early adolescence, its impact cannot be ignored.

Most parents generally believe that once their children enter university and reach adulthood, there is no longer a need for them to invest effort in supervision or guidance. As a result, students often experience an abrupt disconnection from parental educational at the beginning of their university life, which frequently leads to mental health issues and learning difficulties. However, the influence of parental educational on children should be lifelong, differing only in the methods of guidance and the content of involvement at various developmental stages. Therefore, after their children enter university, parents should not withdraw entirely from their educational responsibilities or attribute all educational matters solely to the students themselves or the institution. Particularly during the first year of college, students face substantial changes in their living environment, academic context, and interpersonal relationships. In this critical transition period, parents’ emotional and cognitive involvement can effectively help their children successfully adapt to university life (Smojver-Azic et al., 2016), thereby enabling them to engage more effectively and promptly in academic activities. Consequently, parental educational involvement exerts an important and positive influence on first-year students’ learning engagement.

### 4.3 The mediating role of academic self-efficacy between parental educational involvement and learning engagement

The study also found that parental educational involvement significantly and positively predicted first-year students’ academic self-efficacy, which in turn positively influenced their learning engagement, thereby confirming Hypothesis 2. Bandura’s Social Cognitive Theory posits that social environments such as the family and school exert an important influence on students’ self-efficacy, and the findings of this study support this theoretical proposition. Through educational involvement, parents provide social support for first-year students, enabling them to face academic challenges with confidence, enhance their self-regulatory ability in learning, and improve their academic self-efficacy, which subsequently fosters stronger learning engagement (Turner et al., 2009).

The positive influence of academic self-efficacy on learning engagement is indisputable and has been confirmed by numerous empirical studies (Chemers et al., 2001; Choi, 2005). The higher students’ expectations for their academic outcomes and self-efficacy beliefs, the more likely they are to develop strong learning motivation, which drives them to actively engage in academic activities (Vuong et al., 2010). Therefore, parents who actively participate in their children’s educational by providing emotional support and cognitive guidance can effectively offer autonomy support for first-year college students, thereby stimulating their intrinsic motivation and enhancing their overall academic engagement.

### 4.4 The mediating role of professional identity between parental educational involvement and learning engagement

The study also found that parental educational involvement can significantly and positively predict first-year college students’ professional identity, and further positively influence their learning engagement, thereby verifying Hypothesis 3. The choice of academic major among college students largely originates from parental guidance. After entering university, when first-year students are confronted with the discrepancy between the actual characteristics of their major and their initial expectations, parental guidance at this stage can help students readjust their understanding of the major, adapt to professional learning, and strengthen their professional identity. When students gradually develop recognition and acceptance of the major they study, they naturally exhibit greater enthusiasm for learning and increase their level of engagement in professional study.

### 4.5 The chain mediating role of academic self-efficacy and professional identity between parental educational involvement and learning engagement

The results of the study showed that parental educational involvement positively predicted academic self-efficacy, and by enhancing professional identity, further positively predicted first-year college students’ learning engagement, thereby confirming Hypothesis 4. The effect size of this chain mediation accounted for 57.54% of the total effect, which was higher than the direct effect of parental educational involvement on learning engagement. This finding indicates that parents, by providing positive emotional support and cognitive guidance, offer students greater autonomy support, helping them improve both academic self-efficacy and professional identity, which produces a stronger impact on learning engagement than direct parental influence alone.

This result also aligns with the developmental characteristics of university students. During early adulthood, students’ sense of autonomy and independence develops rapidly. Direct parental preaching or control may lead to resistance or emotional opposition from their children. In contrast, when parental influence is mediated through students’ internal factors, such as self-efficacy and professional identity, it tends to yield more effective and sustainable outcomes in promoting learning engagement.

### 4.6 Implications and limitations

The findings of this study indicate that moderate parental educational involvement not only directly enhances first-year college students’ vigor, dedication, and absorption in learning but also indirectly influences their learning engagement through the chain mediating effects of academic self-efficacy and professional identity. The results confirm the impact of parental educational involvement during early adulthood, breaking conventional stereotypes and offering important insights for guiding family educational at this developmental stage.

The implications can be summarized in three key points. First, family educational during early adulthood remains equally important, and parents should not relinquish their role in value guidance for college students. Second, the degree and manner of parental educational involvement should be adjusted in accordance with students’ developmental and psychological needs. Unlike the directive and didactic approaches appropriate during adolescence, parental involvement at this stage should emphasize emotional support and value-oriented guidance. Third, in the context of spatial and temporal separation between parents and college students and the enhanced autonomy characteristic of university life, parents can indirectly influence students’ learning engagement by fostering their academic self-efficacy and professional identity.

However, the study also has several limitations. First, the research sample was drawn from a single local undergraduate university, making it difficult to determine whether the relationships among variables hold true for other types of higher educational institutions. Second, the study focused solely on first-year students, so it remains unclear whether the observed effects apply to students in other academic years. Third, the research employed a cross-sectional design, which limits the ability to examine whether the influence of parental educational involvement on learning engagement is sustained over time or specific to the unique transitional period of the first year. Future studies should improve the research design, broaden the sample scope, and adopt longitudinal methods to further explore the long-term effects of parental educational involvement on college students’ learning engagement.

## 5 Conclusion

In conclusion, this study shows that parental educational involvement enhances first-year students’ learning engagement not only directly, but also indirectly through three empirically supported pathways: (1) academic self-efficacy, (2) professional identity, and (3) the sequential mechanism from academic self-efficacy to professional identity. These results can be translated into implementable teaching and training activities by aligning intervention content with each pathway. To target the academic self-efficacy pathway, first-year courses should incorporate structured mastery experiences (e.g., low-stakes diagnostic tasks, staged assignments, and frequent formative feedback) that help students build confidence in managing academic demands, thereby sustaining engagement. To activate the professional identity pathway, departments can embed identity-based learning activities such as discipline orientation modules, faculty role-model sharing, and reflective tasks that connect course learning to future professional roles, strengthening students’ sense of purpose and commitment to their field. Importantly, because the findings support a chain mediation, institutions may adopt a sequenced first-year program that begins with efficacy-building supports in the early semester and subsequently deepens identity development through mentoring, career-linked projects, and structured reflection, so that increased competence beliefs are converted into stronger professional identification and, ultimately, higher learning engagement. In parallel, parent-facing guidance can promote developmentally appropriate educational involvement (goal support, encouragement, and resource provision) that reinforces students’ self-efficacy and professional identity without undermining autonomy.

## Data Availability

The raw data supporting the conclusions of this article will be made available by the authors, without undue reservation.
